# Laboratory Diagnosis of SARS

**DOI:** 10.3201/eid1005.030682

**Published:** 2004-05

**Authors:** Paul K. S. Chan, Wing-Kin To, King-Cheung Ng, Rebecca K. Y. Lam, Tak-Keung Ng, Rickjason C. W. Chan, Alan Wu, Wai-Cho Yu, Nelson Lee, David S. C. Hui, Sik-To Lai, Ellis K. L. Hon, Chi-Kong Li, Joseph J. Y. Sung, John S. Tam

**Affiliations:** * The Chinese University of Hong Kong, Prince of Wales Hospital, Shatin, New Territories, Hong Kong SAR, China; † Princess Margaret Hospital, Kowloon, Hong Kong SAR, China

**Keywords:** coronavirus, diagnosis, reverse-transcription–polymerase chain reaction, SARS, virus isolation, viral shedding

## Abstract

The virologic test results of 415 patients with severe acute respiratory syndrome (SARS) were examined. The peak detection rate for SARS-associated coronavirus occurred at week 2 after illness onset for respiratory specimens, at weeks 2 to 3 for stool or rectal swab specimens, and at week 4 for urine specimens. The latest stool sample that was positive by reverse transcription–polymerase chain reaction (RT-PCR) was collected on day 75 while the patient was receiving intensive care. Tracheal aspirate and stool samples had a higher diagnostic yield (RT-PCR average positive rate for first 2 weeks: 66.7% and 56.5%, respectively). Pooled throat and nasal swabs, rectal swab, nasal swab, throat swab, and nasopharyngeal aspirate specimens provided a moderate yield (29.7%–40.0%), whereas throat washing and urine specimens showed a lower yield (17.3% and 4.5%). The collection procedures for stool and pooled nasal and throat swab specimens were the least likely to transmit infection, and the combination gave the highest yield for coronavirus detection by RT-PCR. Positive virologic test results in patient groups were associated with mechanical ventilation or death (p < 0.001), suggesting a correlation between viral load and disease severity.

Severe acute respiratory syndrome (SARS) is a new human disease caused by a novel coronavirus, SARS-associated coronavirus (SARS-CoV) (*1**–**5*). In Hong Kong, the first recognized outbreak of SARS occurred in early March 2003 in the Prince of Wales Hospital (*6**,**7*). Subsequently, outbreaks were reported from other hospitals and from the community (*8*). As of September 26, 2003, 8,098 cases had been reported to the World Health Organization (WHO) from 29 cities, 1,755 of which were found in Hong Kong (*9*). No new cases have been found in Hong Kong since June 11, 2003, and on June 23, 2003, WHO removed Hong Kong from the list of areas with local transmission (*10*). Since identification of the culprit virus in late March 2003, a network has been set up in Hong Kong to provide centralized laboratory diagnostic services for patients with suspected cases of SARS. The diagnostic approach was based on a combination of serologic testing, reverse transcription–polymerase chain reaction (RT-PCR), and virus isolation. Here, we report our experience with the laboratory diagnosis for SARS-CoV infection during this outbreak in Hong Kong, with emphases on the viral shedding pattern, the diagnostic yield of various specimen types, and detection methods.

## Materials and Methods

### Patients

This retrospective study analyzed laboratory records of patients admitted to six public hospitals in Hong Kong during the SARS epidemic from March to June 2003. The first inclusion criterion was serologic evidence of SARS-CoV infection. Altogether, 433 patients who exhibited either seroconversion or a fourfold rise in anti–SARS-CoV immunoglobulin (Ig)G antibody titer were identified. Detection of anti-SARS-CoV IgG antibody was based on an in-house immunofluorescence assay that used virus-infected cells. Of the 433 patients with positive serologic test results, 18 were excluded because no samples had been collected for virus detection. As a result, 415 patients were included in this study. Twelve were pediatric patients 3–16 years of age (mean 11.3, standard deviation [SD] 4.1), divided equally between girls and boys. Three hundred thirty-five were adult patients 17–64 years of age (mean 37.1, [SD 11.2), with 60.9% females. The remaining 68 were elderly patients 65–97 years of age (mean 76.7, SD 8.2), with 37 (54.4%) women. Altogether, 48/335 (14.3%) of the adult group and 2/68 (2.9%), respectively, of the elderly group required ventilation and received intensive care but recovered; 4/335 (1.2%) of adults and 21/68 (30.9%) of elderly patients died of the infection. All children recovered without requiring mechanical ventilation or intensive care.

### Specimen Collection

Respiratory, stool, and rectal swab specimens were collected in viral transport medium, and urine samples were transported in sterile containers. For some patients, throat and nasal swab samples were pooled into a single specimen container and processed as a single specimen. These samples were referred as “pooled throat and nasal swabs” for the purpose of analysis in this study. Specimens collected were refrigerated (approximately 10°C) until delivery, which were done on the same day in most circumstances. Specimens were kept in iceboxes during delivery to the designated centralized laboratory. SARS-CoV investigations were performed on fresh specimens without prior freezing and thawing.

### Viral RNA Detection

SARS-CoV detection by RT-PCR was conducted in two laboratories based on the same primer set COR-1 (sense) 5′ CAC CGT TTC TAC AGG TTA GCT AAC GA 3′, and COR-2 (antisense) 5′ AAA TGT TTA CGC AGG TAA GCG TAA AA 3′ (*11*). The specimens were centrifuged at 10,000 x *g* for 1 min, and 140 μL of the supernatant were used for RNA extraction using the QIAamp viral RNA Mini Kit (QIAGEN, Hilden, Germany) according to the manufacturer’s protocol. Reverse transcription of RNA was conducted in a 20-μL reaction mix containing 4.2 μL of extracted RNA preparation, 2.5 μmol/L of random hexamer and 50 U of reverse transcriptase (Applied Biosystems, Foster City, CA). After incubation at room temperature for 10 min and at 42°C for 30 min, the reaction was stopped by heating at 95°C for 5 min and chilled on ice. The subsequent PCR was conducted in a 50-μL reaction mix containing 5 μL of cDNA template, 1 μmol/L of each primer COR-1 and COR-2, 1.5 U Taq polymerase (Amersham Biosciences, Uppsala, Sweden), 0.2 mmol/L of each deoxynucleoside triphosphate and 2.0 mmol/L magnesium chloride. The cycling conditions were 94°C for 3 min; 45 cycles of 94°C for 30 sec, 60°C for 30 sec, and 72°C for 1 min, and a final extension at 72°C for 7 min. The PCR amplicons were visualized by ethidium bromide staining after agarose gel electrophoresis.

The same RNA extraction method was used in laboratory B. The RT-PCR was carried out in a single-tube system (Superscript One-Step RT-PCR with Platinum Taq, Invitrogen, Carlsbad, CA), in a 25-μL reaction mix containing 0.6 μmol/L of each COR-1 and COR-2 primer, 0.2 mmol/L of each deoxynucleoside triphosphate and 1.2 mmol/L magnesium sulphate. The reverse transcription was conducted at 54°C for 30 min. After the mixture was held at 94°C for 3 min, it underwent 45 cycles of amplification at 94°C for 45 sec, 60^o^C for 45 sec, 72°C for 45 sec, and final extension at 72°C for 7 min. The PCR amplicon was also detected by agarose gel electrophoresis as in laboratory A.

All reagent preparation, sample extraction, amplification, and amplicon detection procedures were conducted in separate areas and under strengthened precautions to avoid cross-contamination. The lower detection limit of the RT-PCR assays was determined by testing preparations with known copies of SARS-CoV as determined by real-time RT-PCR. Both laboratories showed a lower detection limit of 50 viral copies per reaction. In all test runs, positive controls containing approximately 100 copies of viral RNA in viral transport medium were included, and double distilled water was used as a negative control. Positive samples were confirmed by repeating the RNA extraction and RT-PCR from the original samples.

### Virus Isolation

Virus isolation for SARS-CoV was performed in laboratory B. Specimens were injected into African green monkey (Vero E6) cell monolayers. For stool or rectal swab samples, the suspension was passed through a 0.45-μm filter before injection. The cell culture tubes were examined daily for diffuse, refractile, rounding cytopathic effects characteristic of SARS-CoV. When cytopathic effects were observed, the cells were stained by the indirect immunofluorescence technique with a convalescent-phase serum sample collected from a SARS patient. The identity of the isolate was further confirmed by RT-PCR.

### Statistical Analysis

Statistical tests were performed by using the Statistical Package for the Social Sciences software (SPSS 10.1.0, Inc., Chicago, IL). The chi-square test was used to analyze categorical variables. All statistical tests were two-tailed and p values ≤0.05 were regarded as significant.

## Results

### Specimen Profile

**Figure 1:**
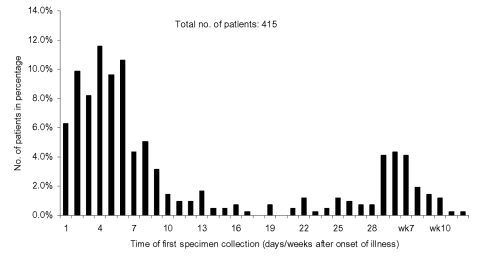
Time of first specimen collection.

Altogether, 624 respiratory specimens, 671 stool or rectal swab specimens, and 314 urine specimens were collected from the 415 study patients for RT-PCR; 738 respiratory, 810 stool or rectal swab, and 531 urine specimens were submitted for virus isolation; and 558 respiratory, 318 stool or rectal swab, and 296 urine specimens were tested by both RT-PCR and virus isolation. The mean number of specimens collected from each patient was 5.3 (range 1–32, SD 5.1). The mean time of collection of the first specimen was 13.5 days (range 1–88, SD 16.5) after the onset of symptoms (Figure 1). Patients whose first specimens were collected at a later stage of illness had become ill early in the outbreak when no diagnostic test was available.

### Shedding Profile

**Figure 2:**
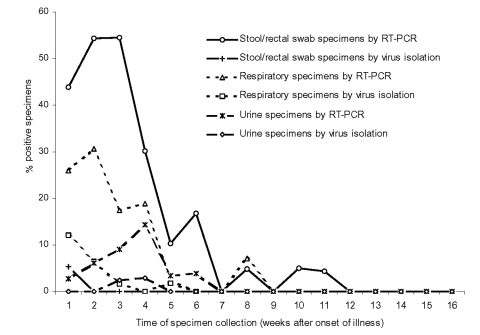
Positive rates of specimen groups according to time of collection from onset of symptoms. The number of specimens tested is shown in Table 1.

To analyze the profile of viral shedding, specimens were grouped into categories: respiratory, stool or rectal swab, and urine. Respiratory specimens included tracheal aspirate, nasopharyngeal aspirate, throat swab, throat washing, nasal swab, and pooled throat and nasal swabs. The viral shedding profile is shown in Figure 2; the number of specimens tested is shown in Table 1. Stool/rectal swab specimens provided the highest positive rate by RT-PCR, followed by respiratory and urine specimens. The RT-PCR positive rate for respiratory specimens increased slightly from week 1 to week 2 after the onset of illness and then dropped to lower levels at week 3 and week 4. The positive rate for stool/rectal swab peaked at week 2 and week 3 and then dropped sharply. The positive rate for urine specimens increased gradually and peaked at week 4. Viral shedding beyond week 6 was rare, with only three stool samples (collected on day 54, day 67, and day 75, respectively) and one respiratory sample collected on day 50 positive by RT-PCR. As for virus isolation, the latest positive specimen was collected on day 31 for a respiratory specimen, day 23 for a urine specimen, and day 6 for a stool sample.

**Table 1:** Specimens tested after onset of illness

Collection time (wk)	Specimen type					
	Stool/rectal swab		Respiratory		Urine	
	RT-PCR^a^	Virus isolation	RT-PCR	Virus isolation	RT-PCR	Virus isolation
1	32	38	243	280	75	110
2	35	40	134	153	82	86
3	44	84	57	62	33	41
4	43	92	37	36	21	35
5	96	113	41	57	29	64
6	110	123	30	50	26	72
7	80	84	18	30	9	38
8	54	55	14	22	10	33
9	49	52	16	27	6	26
10	34	35	16	12	9	10
11	44	44	10	5	9	11
12	21	21	4	2	2	3
13	16	16	2	1	2	1
14	7	7	2	1	1	1
15	5	5	0	0	0	0
16	1	1	0	0	0	0

^a^RT-PCR, reverse transcription–polymerase chain reaction.

**Figure 3:**
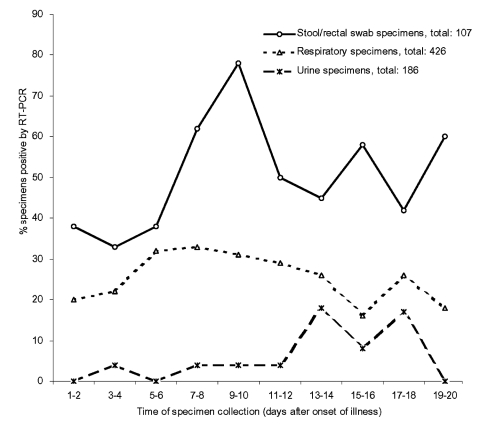
Positive rates of specimens collected within the first 3 weeks.

The RT-PCR results of specimens collected within the first 3 weeks after the onset of illness were analyzed to further clarify the viral shedding pattern. The positive rate for respiratory specimens began to increase on day 5 and remained high during the second week. The positive rate for stool/rectal swab specimens peaked at days 9 and 10 and remained high during the second and third week, whereas the detection rate for urine specimens started to increase at the end of the second week (Figure 3).

### Diagnostic Yield

The RT-PCR and virus isolation results of different specimen types collected within the first 4 weeks after the onset of symptoms are shown in Table 2. When RT-PCR was used for virus detection, tracheal aspirate and stool provided a high diagnostic yield, with an average positive rate of 66.7% and 56.5%, respectively, for the first 2 weeks. Pooled throat and nasal swabs, rectal swab, nasal swab, throat swab and nasopharyngeal aspirate provided a moderate yield with average positive rates ranging from 29.7% to 40.0% for the first 2 weeks, whereas throat washing and urine specimens provided a lower yield with an average positive rate of 17.3% and 4.5%, respectively. The yield from virus isolation was much lower than from RT-PCR, and no specimen was positive by culture but negative by RT-PCR.

**Table 2:** Diagnostic yield of specimen types to detect SARS-CoV according to time of collection^a^

	No. positive specimens/no. tested for SARS-CoV (%)						
	RT-PCR				Virus isolation		
Specimen type	1 week	2 weeks	3–4 weeks		1 week	2 weeks	3–4 weeks
Respiratory							
Tracheal aspirate	1/2 (50.0)	1/1 (100)	4/4 (100)		2/3 (66.7)	1/1 (100)	0/3
Pooled throat and nasal swabs	6/17 (35.3)	2/3 (66.7)	2/5 (40.0)		4/18 (22.2)	0/3	0/1
Nasal swab	9/27 (33.3)	5/14 (35.7)	1/17 (5.9)		3/29 (10.3)	2/18 (11.1)	0/19
Nasopharyngeal aspirate	39/138 (28.3)	15/44 (34.1)	6/10 (60.0)		23/171 (13.5)	6/54 (11.1)	0/9
Throat swab	5/19 (26.3)	5/14 (35.7)	3/10 (30.0)		2/23 (8.7)	0/15	1/15 (6.7)
Throat washing	4/40 (10.0)	13/58 (22.4)	1/48 (2.1)		0/36	1/62 (1.6)	0/51
Nonrespiratory							
Rectal swab	5/11 (45.5)	2/10 (20.0)	3/7 (42.9)		0/14	0/12	0/35
Stool	9/21 (42.9)	17/25 (68.0)	34/80 (42.5)		2/24 (8.3)	0/28	0/141
Urine	2/75 (2.7)	5/82 (6.1)	6/54 (11.1)		0/110	0/86	2/76 (2.6)

^a^SARS-CoV, severe acute respiratory syndrome–associated coronavirus; RT-PCR, reverse transcription–polymerase chain reaction.

### RT-PCR Versus Isolation

To compare the sensitivity of RT-PCR and virus isolation for detecting SARS-CoV, a subgroup analysis was performed on 1,172 specimens that had been submitted for both RT-PCR and virus isolation. The isolation/RT-PCR index, defined as the number of isolation-positive specimens per RT-PCR-positive specimens, was highest for respiratory samples, particularly for pooled throat and nasal swabs, tracheal aspirate, and nasopharyngeal aspirate. The isolation/RT-PCR index for stool or rectal swab samples was approximately 5- to 10-fold lower when compared with that for respiratory specimens (Table 3).

**Table 3:** Comparison on positive rates of RT-PCR and virus isolation^a^

Specimen type (no.)	No. (%) of specimens tested positive^b^		Isolation/RT-PCR index^c^
	RT-PCR	Virus isolation	
Pooled throat and nasal swab (30)	8 (26.7)	4 (13.3)	0.50
Tracheal aspirate (13)	6 (46.2)	2 (15.4)	0.33
Nasopharyngeal aspirate (183)	52 (28.4)	14 (7.7)	0.27
Throat swab (58)	11 (19.0)	2 (3.4)	0.18
Nasal swab (56)	14 (25.0)	2 (3.6)	0.14
Urine (296)	14 (4.7)	2 (0.7)	0.14
Throat washing (218)	17 (7.8)	1 (0.5)	0.06
Stool (262)	70 (26.7)	2 (0.8)	0.03
Rectal swab (56)	12 (21.4)	0 (0)	0

^a^RT-PCR, reverse transcription–polymerase chain reaction. ^b^Only specimens tested by both RT-PCR and virus isolation are included. ^c^No. of isolation-positive specimens per RT-PCR-positive specimen.

### Positive Rate by Patient

Altogether, 132 (31.8%) of the 415 study patients had SARS-CoV detected by RT-PCR or virus isolation. To analyze factors associated with positive virologic testing results, a subgroup analysis was performed on 342 patients whose first specimens were collected within 4 weeks of illness onset. Within this subgroup, 128 (37.4%) patients had one or more positive results by RT-PCR or virus isolation. The mean number of positive specimens among these patients was 1.8 (range 1–10, SD 1.7). The characteristics of patients with and without positive specimens are shown in Table 4. A higher positive detection rate for SARS-CoV was observed for patients with more severe disease (p < 0.001 by chi-square test).

**Table 4:** Positive rates for SARS-CoV for various patient groups^a^

	SARS-CoV result by RT-PCR/virus isolation	
Patient characteristics (n)^b^	No. (%) of positive patients^c^ (n = 128)	No. (%) of negative patients (n = 214)
Sex		
Female (210)	83 (39.5)	127 (60.5)
Male (132)	45 (34.1)	87 (65.9)
Age group (years)		
≤16 (8)	4 (50.0)	4 (50.0)
17–64 (271)	96 (35.4)	175 (64.6)
≥65 (63)	28 (44.4)	35 (55.6)
No. of specimens tested		
1–2 (116)	39 (33.6)	77 (66.4)
3–5 (111)	36 (32.4)	75 (67.6)
≥6 (115)	53 (46.1)	62 (53.9)
Time of first specimen collected (weeks after illness onset)		
1 (251)	97 (38.6)	154 (61.6)
2 (57)	21 (36.8)	36 (63.2)
3 (11)	4 (36.4)	7 (63.6)
4 (23)	6 (26.1)	17 (73.9)
Disease outcome		
Recovered, not requiring ventilation or intensive care (279)	91 (32.6)	188 (67.4)
Recovered after ventilation or intensive care (40)	22 (55.0)	18 (45.0)
Died (23)	15 (65.2)	8 (34.8)

^a^SARS-CoV, severe acute respiratory syndrome–associated coronavirus. ^b^Only patients with their first specimens collected within 4 weeks of onset of illness are included ^c^Patients with one or more specimen(s) positive for SARS-CoV by RT-PCR and/or virus isolation.

## Discussion

Identifying the causal agent of the novel emerging infection, SARS, shortly after recognizing its spread in humans, was a remarkable medical accomplishment. This achievement led to the hope for an accurate laboratory diagnosis to guide patient management and to control the spread of infection. During the course of the outbreak, a few centralized laboratories were set up in Hong Kong. All possible resources were deployed to provide a rapid diagnostic service for SARS patients, and a turnaround time of 24 to 48 hours was achieved for RT-PCR. From our experience, more than half of the patients did not have any positive virologic findings. For these patients, the diagnosis could not be confirmed until a convalescent-phase serum specimen was available at a later stage. Thoroughly understanding the viral shedding pattern, the diagnostic yield of various specimen types, and various detection methods is crucial to improve the diagnostic performance.

For most acute respiratory viral infections, the maximal viral shedding occurs in the first few days after illness onset and seldom lasts for more than 10 days (*12**–**14*). However, our data indicated that respiratory shedding of SARS-CoV increased over the first week and remained high during the second week. In addition, respiratory shedding >2 weeks after the onset of symptoms was common. This pattern of respiratory shedding is consistent with a previous report of a community outbreak in Hong Kong (*15*). We found that the peak of viral shedding in stool occurred a few days after that of respiratory shedding. The ability to detect virus in stool specimens peaked at the beginning of the second week and remained high over week 3 and week 4. Occasionally, the shedding of virus in stool could last for more than 6 weeks after the onset of symptoms. The viral shedding peak in urine occurred even later, at weeks 3–4.

In summary, viral shedding of SARS-CoV peaks at a time later than expected and occurs when patients are being hospitalized. This, together with the prolonged viral shedding, could partly explain the propensity for this infection to be transmitted in healthcare settings. We observed that all those who shed virus for a prolonged period (arbitrarily defined as the shedding viruses >6 weeks after onset of symptoms) had their positive samples collected while still critically ill and had received intensive care. The infectiousness of these patients is difficult to discuss because the virus was detected by RT-PCR but not by virus isolation. Nevertheless, further investigations on whether the adverse outcome could be related to inadequate viral clearance are worth pursuing.

Available data that compare the diagnostic yield of various specimen types are still limited. Wu et al. found that virus was detected in 73% (49/67) of liquid nasopharyngeal gargling samples by a fluorescent PCR (*16*). However, our data showed that throat washing samples were the most inferior respiratory specimens. In addition to the difference in the sensitivity of detection assays used, the procedures of gargle sample collection could have affected the diagnostic yield. Yam et al. reported that nasopharyngeal aspirate specimens collected between days 1 and 5 after admission provided a similar diagnostic yield when compared to stool samples collected between days 5 and 10 (*17*). However, data comparing respiratory and stool specimens collected at the same period were not available in their study. In an investigation on a community outbreak in Hong Kong, Peiris et al. reported that respiratory viral shedding peaked during the second week (*15*). A high positive rate was also obtained from stool samples collected during the second week, but the yield for first week stool samples was not available for comparison.

Nasopharyngeal aspirate is generally regarded as the specimen of choice for detecting respiratory viruses. However, for SARS, the great risk of generating infectious aerosols during the aspiration procedure needs to be considered. We found that pooled throat and nasal swab specimens provided a higher diagnostic yield compared with nasopharyngeal aspirates. Our data indicate that a combination of stool sample and pooled throat and nasal swab specimens should be the specimens of choice for a safe and high-yield SARS-CoV detection. In situations where specimen load is high, pooling of stool sample with throat and nasal swabs for RT-PCR can be considered to minimize the reagent and personnel costs.

SARS-CoV was first isolated from a monkey kidney cell line and is known to produce characteristic cytopathic effects after a few days of incubation in Vero or Vero E6 cell monolayers. At present, the ideal in vitro growth conditions have not yet been elucidated. Our data on isolation/RT-PCR index showed that about 10%–50% of the RT-PCR–positive respiratory and urine specimens had virus grown from Vero E6 cell culture. However, stool and rectal swab specimens had a much lower isolation/RT-PCR index. The presence of toxic substances in stool or rectal swab samples may have interfered with virus isolation. However, toxicity was only occasionally observed on Vero E6 monolayers after adding stool or rectal swab samples. SARS-CoV can survive for at least 2–4 days at room temperature when mixed with diarrheal or normal stool specimens (*18*). Thus, the poor isolation rate could not be a result of viral inactivation by fecal contents during specimen storage and transport. The big difference in isolation rate from stool compared to respiratory and urine samples deserve further investigation, and the possibility of viral growth interference due the presence of immunoglobulin (Ig)A antibodies needs to be considered.

We found that positive virologic results were associated with more adverse outcomes in patients. This observation could be confounded by the fact that only high-yield specimens, e.g., tracheal aspirate, could be obtained from intubated patients. We verified this point by examining the results of testing other samples from patients with viruses detected from tracheal aspirate samples. We found that all except one of these patients also had viruses detected from other specimen types. Thus, our observations are in line with the fact that more severely affected patients shed a higher load of virus, which facilitated the detection of the virus.

Several options could be considered to improve the ability to accurately diagnose SARS-CoV infection. First, levels of viremia should be included in the diagnostic algorithm because we have found SARS-CoV RNA from blood samples taken within the first few days of onset of symptoms. If this approach is successful, it will close the gap caused by lower virus shedding from the gastrointestinal or respiratory tract that occurs in the first few days after the onset of symptoms. Second, a SARS-CoV-specific monoclonal antibody would be valuable in developing an immunofluorescence assay to detect virus-infected cells from respiratory samples. Such an approach has been shown to provide high sensitivity for influenza and respiratory syncytial viruses. Third, an assay should be developed to detect viral antigens from stool samples as is available for rotavirus detection. Further work to improve the sensitivity and specificity of diagnostic assays for SARS-CoV is needed. The unusual shedding pattern of SARS-CoV should be considered when formulating infection control strategies.
